# Aquatic Therapy after Incomplete Spinal Cord Injury: Gait Initiation Analysis Using Inertial Sensors

**DOI:** 10.3390/ijerph191811568

**Published:** 2022-09-14

**Authors:** Silvia Fantozzi, Davide Borra, Matteo Cortesi, Alberto Ferrari, Simone Ciacci, Lorenzo Chiari, Ilaria Baroncini

**Affiliations:** 1 Department of Electrical, Electronic, and Information Engineering “Guglielmo Marconi”, University of Bologna, Viale Risorgimento 2, 40136 Bologna, Italy; 2 Health Sciences and Technologies—Interdepartmental Centre for Industrial Research, University of Bologna, Viale Risorgimento 2, 40136 Bologna, Italy; 3 Department for Life Quality Studies, University of Bologna, Via del Pilastro 8, 40126 Bologna, Italy; 4 Department Biomedical and Neuromotor Sciences, University of Bologna, Via del Pilastro 8, 40126 Bologna, Italy; 5 Montecatone Rehabilitation Institute S.p.A., Via Montecatone 37, 40026 Imola, Italy

**Keywords:** anticipatory postural adjustment, inertial measurement unit (IMU), wearable sensors, water activity, first step

## Abstract

Populations with potential damage to somatosensory, vestibular, and visual systems or poor motor control are often studied during gait initiation. Aquatic activity has shown to benefit the functional capacity of incomplete spinal cord injury (iSCI) patients. The present study aimed to evaluate gait initiation in iSCI patients using an easy-to-use protocol employing four wearable inertial sensors. Temporal and acceleration-based anticipatory postural adjustment measures were computed and compared between dry-land and water immersion conditions in 10 iSCI patients. In the aquatic condition, an increased first step duration (median value of 1.44 s vs. 0.70 s in dry-land conditions) and decreased root mean squared accelerations for the upper trunk (0.39 m/s^2^ vs. 0.72 m/s^2^ in dry-land conditions) and lower trunk (0.41 m/s^2^ vs. 0.85 m/s^2^ in dry-land conditions) were found in the medio-lateral and antero-posterior direction, respectively. The estimation of these parameters, routinely during a therapy session, can provide important information regarding different control strategies adopted in different environments.

## 1. Introduction

Among the functional tasks for evaluating balance control, gait initiation was found to be particularly revealing for identifying motor disorders in populations with specific impairments of the postural control system (sensory or motor deficits) or fear of falling [1,2,3]. Gait initiation is defined as the transition from stationary standing to steady-state walking [4]. Involving a modification of the dimension of the support base and, simultaneously, a progression of the center of mass, gait initiation is classically used in the literature to investigate how the central nervous system controls balance during a whole-body movement. Indeed, this motor task requires integrating multiple kinds of sensory information (somatosensory, vestibular, and visual) and the coordination of multiple skeletal muscles (from lower limbs to the trunk).

Gait initiation is typically divided into two phases: the anticipatory postural adjustment (APA) phase, related to the motor and cognitive components of movement preparation, and the execution of the first step, related to movement realization [1,5]. With aging or pathologies affecting the locomotor apparatus, the reduced capacity to use the different resources involved in balance control increases the risk of falling [6,7]. Populations with potential damage to any of the motor control subsystems have been studied during the specific execution of gait initiation [1]: elderly or impaired subjects suffering from Parkinson′s disease [8,9,10], stroke [11], and incomplete spinal cord injury (iSCI) [12,13]. Several specific motor task disturbances were observed in individuals with Parkinson′s disease by many authors: a delayed release of APA, hypokinetic (reduced scaling), and bradykinetic (abnormal timing) APA [8]. Conversely, few studies analyzed the gait initiation after iSCI, finding compromised postural stability, such as longer gait initiation time and reduced first step length [13]. The single-support and the bipedal phases were found to be the most challenging phases for the dynamic and the postural component, respectively [12]. Further investigations should be performed to address applications of therapeutic interventions for iSCI.

Among physical therapies aiming at improving gait initiation, the aquatic one is widely used in neurorehabilitation, as it provides (i) a reduction in apparent body weight due to buoyant force, (ii) a variation in muscle action due to water resistance, and (iii) an increased commitment in balance control due to the stimuli associated with water turbulence when moving in the pool [14]. A systematic review found “fair” evidence for aquatic therapy towards increasing dynamic balance and improving gait speed [15]. Regarding iSCI patients, aquatic activity effectively benefits participants’ functional capacity [16]. Aquatic exercises of passive/active movements and walking in water were effective in decreasing the energy cost of level walking at a low speed of progression [17]. A case series analyzing quasi-static standing balance during water immersion after iSCI found an increased center of pressure sway and upper to lower trunk acceleration ratios in the antero-posterior (AP) direction with respect to dry-land conditions, reflecting the balance and sensorimotor impairment of the participants [18]. Specifically, during gait initiation in water immersion, a few individuals showed a faster APA and slower execution phase compared to dry land, together with a different trunk control strategy [19]. In both studies, participants perceived the motor task to be more challenging in water but safer than on dry land. These preliminary results suggest that a deeper and more extensive investigation of gait initiation performed by iSCI patients during aquatic therapy is required, as it can give fundamental indications for rehabilitation treatments for this specific condition.

Gait initiation features have been typically characterized using electromyography, force plates, and camera-based systems in gait analysis laboratories. Recently, inertial measurement units (IMUs) were exploited for the same purpose [9,20,21,22]. Wearable sensors offer novel possibilities for ambulatory employment with significant clinical practice benefits. They are portable and easy-to-use tools that can be employed to monitor the effects of disease progression, interventions, and therapies such as aquatic therapy. Furthermore, IMUs can be easily waterproofed, in contrast with other instrumentations typically used for gait initiation evaluation.

The aim of the present study was to compare gait initiation in iSCI patients between two conditions, dry land and water immersion, using wearable IMUs. We hypothesized that the different environment would enhance different motor control strategies in iSCI populations. Temporal and acceleration-based (at the lower and upper trunk levels) APA measures were computed and compared between the two environments. The final intention is to propose an easy-to-use protocol for evaluating gait initiation during aquatic therapy sessions.

## 2. Materials and Methods

### 2.1. Participants

Adult participants with iSCI were recruited at the Montecatone Rehabilitation Institute. Inclusion criteria were (i) over 18 and below 70 years of age with traumatic or non-traumatic etiology of iSCI; (ii) incomplete motor lesion associated with any neurological level of injury, scored as D according to the ASIA classification [23]; (iii) with the damaging event occurred longer than 12 months before evaluations; (iv) using walking as a prevalent system of locomotion; and (v) with intact cognitive skills. Exclusion criteria were (i) the presence of orthopedic, metabolic, cardiovascular, or pain conditions that contraindicate the performance of the functional assessment tests employed in the study, and (ii) pregnancy.

Ten iSCI patients (1 female and 9 males, age 65 ± 8 years, mass 82.8 ± 15.3 kg, height 173.8 ± 7.1 cm) were recruited. Neurological levels of the lesions were from L5 to C6. The mean and standard deviation of clinical scores were (i) American Spinal Injury Association Lower Extremity Motor Score: 21.9 ± 4.3 and 20.7 ± 6.3 for right and left limbs, respectively; (ii) WISCII [24]: 18.4 ± 2.6; (iii) Ashworth scale [25]: 1.1 ± 1.2.

### 2.2. Experimental Procedure

Participants performed gait initiation on land and in water on the same day. Participants stood in the same comfortable standing position and were instructed to initiate gait using their preferred leg. The functional trial task was performed in the aquatic therapy pool of the Montecatone Rehabilitation Institute (120 cm depth at a temperature of 30 °C), and for dry-land conditions, in the area outside the therapy pool.

**Figure 1:**
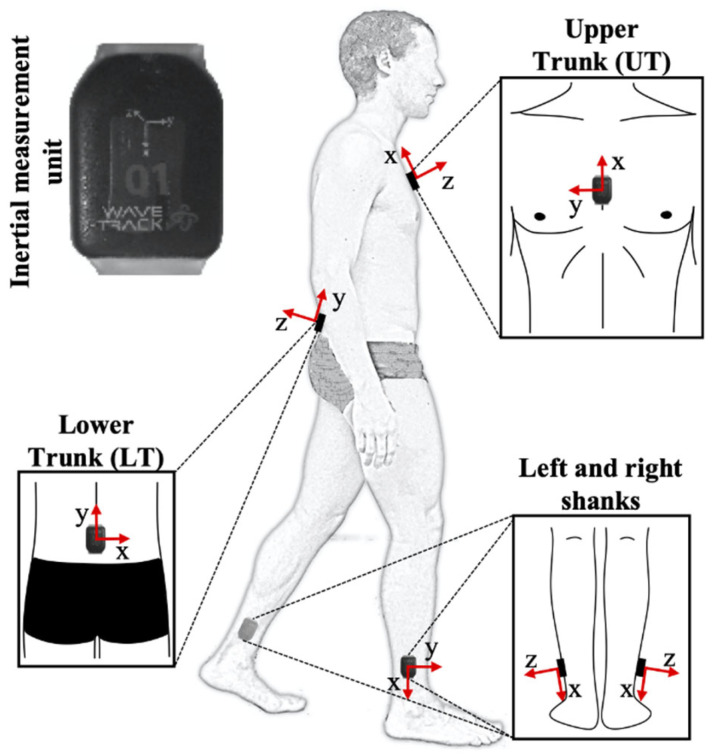
Shape of natively waterproofed inertial measurement units (IMUs) and positioning of IMU sensors on lower trunk, upper trunk, and shanks. Alignment of axes (X, Y, and Z) of reference system are shown for each sensor.

Four waterproof IMUs (WaveTrack, Cometa, Milano, accelerometer sensitivity: 1563 mV/g; full scale: ±8 g; gyroscope sensitivity: 1.3 mV/g; full scale: ±1000°/s, sampling frequency: 285 Hz) were positioned on the right and left lower parts of the shanks, the lower trunk (LT), and on the upper trunk (UT) (Figure 1). IMU sensors of the shanks were aligned with the X-axis to the long axis of the fibula pointing distally (within the distal third, close to the lateral malleolus). The IMU of the LT had the X-axis oriented along the line linking the posterior superior iliac spines pointing towards the right. Lastly, the IMU of the UT was positioned over the flat portion of the sternum, with the Z-axis pointing away from the body and the X-axis pointing cranially.

### 2.3. Data Analysis

The algorithm proposed by Mancini et al. [20] was used to identify the APA onset and APA offset from IMU-based measurements of each recording trial in dry-land and water immersion conditions. In particular, the algorithm exploits the LT acceleration and the shank angular velocities in the medio-lateral (ML) direction. At first, acceleration signals were transformed to a horizontal–vertical coordinate system (alignment of sensor axes to gravity during static recordings) and low-pass filtered using a zero-phase Butterworth filter with a 3.5 Hz cut-off frequency, as performed in [20]. Then, local peaks in the absolute value of LT acceleration were identified (selecting points that are larger than the two adjacent points), and the candidates of APA intervals (each one identified by its own APA onset and offset) were selected as the ones in which the acceleration exceeded a predetermined threshold. For each APA candidate, the threshold was set as 20% of each peak, and only APA candidates lasting at least 0.1 s and at most 1.5 s were considered. The threshold value and minimum and maximum APA duration values were set as in the original implementation [20]. Shank angular velocities were used to identify the toe-off and heel-strike events of the first step by applying the algorithm proposed by Salarian et al. [26]. Lastly, the APA was selected from the APA candidates as the one immediately preceding the toe-off of the first step. Once the APA onset and offset were obtained, the following APA measures, as used in Mancini et al. [20] and Marhino-Bulzelli et al. [19,27], were computed separately for dry-land and water conditions.

i. Temporal measures:
  a. APA duration ($\Delta t_{APA}$), defined as the interval between the APA onset and APA offset.
  b. First step duration ($\Delta t_{1st\;step}$), defined as the interval between the APA offset and the heel-strike of the first step.
ii. Acceleration-based measures during the first step (from APA offset and the heel-strike of the first step):
  a. Root mean squared (RMS) acceleration of the UT in the ML direction (${\mathrm{RMS}}_{ML,\;UT}$).
  b. RMS acceleration of the UT in the AP direction (${\mathrm{RMS}}_{AP,\;UT}$).
  c. RMS acceleration of the LT in the ML direction (${\mathrm{RMS}}_{ML,\;LT}$).
  d. RMS acceleration of the LT in the AP direction (${\mathrm{RMS}}_{AP,\;LT}$).

These APA measures, characterizing each participant, were used to compare gait initiation in dry-land and water conditions.

### 2.4. Statistical Analysis

Descriptive results are presented as mean values ± SD. A one-factor repeated measure MANOVA with a within-subjects design was used to examine the impact of the environment (water vs. land) on dependent variables ($\Delta t_{APA}$, $\Delta t_{1st\;step}$, ${\mathrm{RMS}}_{ML,\;UT}$, ${\mathrm{RMS}}_{AP,\;UT}$, ${\mathrm{RMS}}_{ML,\;LT}$, ${\mathrm{RMS}}_{AP,\;LT}$). Pearson correlation matrices for dependent variables were used to detect the appropriateness of MANOVA statistics. French et al. [28] suggest that moderate correlations are most appropriate for using MANOVA. Our correlations ranged from low to moderate correlation, and MANOVA statistic was applied. The multivariate effects of the dependent variables between environmental conditions were tested using Wilks’ lambda. A significant multivariate effect was followed up with univariate ANOVAs. As only two conditions were investigated for each main effect in the univariate ANOVAs (water and land), post hoc tests of the main effect differences were unnecessary. Effect size (partial eta square, η_p_^2^) was calculated to interpret the meaningfulness of differences and categorized as small (0.01), medium (0.06), or large (0.14) [29]. Statistical significance was set at *p* < 0.05 for all comparisons. All statistical analyses were performed using SPSS version 20.0 (SPSS, Chicago, IL, USA).

## 3. Results

**Figure 2:**
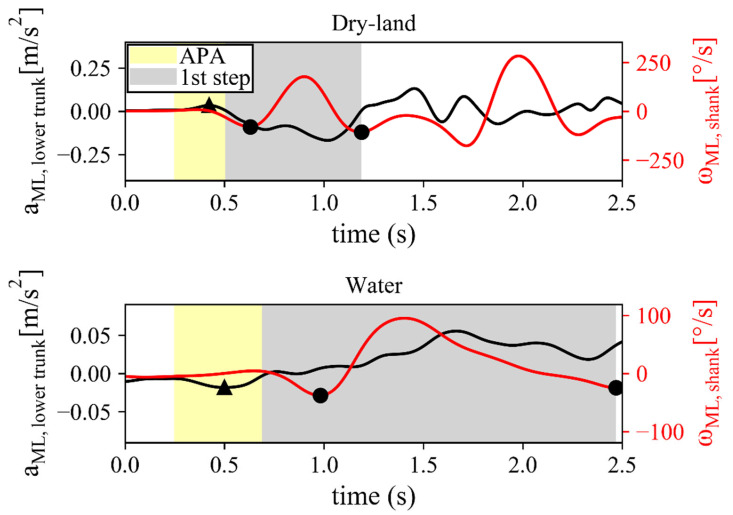
Representative example of the detected APA during gait initiation in dry-land (**upper panel**) and water (**lower panel**) conditions. In each panel, the ML acceleration of the lower trunk (black line) is displayed together with the angular velocity of the shank (red line). Black-filled dots denote toe-off and heel-strike events, while the black-filled triangle denotes the ML acceleration peak. The yellow and gray shaded areas represent the APA and the 1st step intervals, respectively. In the APA interval, the ML acceleration exceeds a threshold of 20% of the peak, while the 1st step interval was defined from APA end to heel-strike.

The patterns of the variables used by the algorithm for detecting APA and the first step duration were similar in water and on dry land. Thus, no difficulties were experienced in identifying the APA temporal events in both environments (see Figure 2 for a representative example in one patient). The inclination of UT in the standing position before the APA was estimated and compared in the two conditions, finding differences < 0.1° (below the degree of uncertainty associated to the device). For this reason, this aspect was considered not to influence the APA measures.

**Figure 3:**
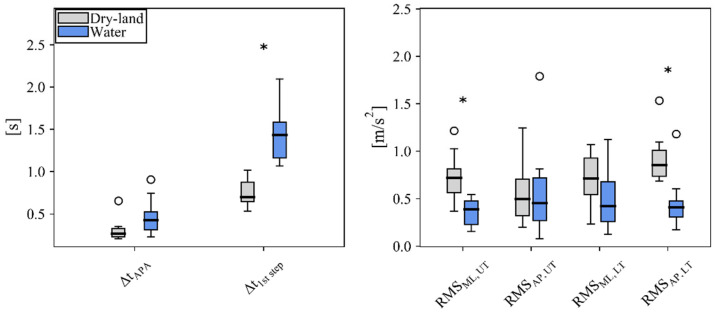
APA measures obtained in dry-land (gray) and water (blue) conditions. The boxplot is reported for each distribution in each panel, where the ticked black line denotes the median value and the circles denote the outliers. Significant differences found between the two conditions (see Section 2.4) are displayed (* *p* < 0.05).

The temporal and acceleration-based variables are reported in Figure 3. The statistical analyses confirmed a significant association between the environmental conditions and gait initiation in iSCI patients: results from the factorial MANOVA showed that environmental conditions significantly affect the combined dependent variables of APA duration, first step duration, and RMS acceleration of UT and LT in AP and ML directions (Wilks’ Λ = 0.070, F6, 4 = 8.912, *p* < 0.05, η_p_^2^ = 0.930).

In the follow-up univariate ANOVAs, the results focus on the main effects for dependent variables. For the main effect of the environmental condition group, significant differences were found in first step duration (F1, 9 = 27.348, *p* < 0.001, η_p_^2^ = 0.752), ${\mathrm{RMS}}_{ML,\;UT}$ (F1, 9 = 24.847, *p* < 0.001, η_p_^2^ = 0.734), and ${\mathrm{RMS}}_{AP,\;LT}$ (F1, 9 = 15.084, *p* < 0.05, η_p_^2^ = 0.626). No other significant mean differences were found for the dependent variables.

However, although not significant, in addition to a slight increase in the APA duration (Figure 3, left panel), it was possible to observe a reduction in the RMS in the ML direction at the LT position in water compared with the dry-land environment (Figure 3, right panel). That is, a reduction in the RMS in the ML direction was observed for both UT and LT positions (even though result was significant only for the UT), overall. Conversely, a different behavior between UT and LT acceleration-based measures in the AP direction emerged: the reduction in the RMS observed for LT was not found for UT, as comparable values were obtained (Figure 3, right panel).

## 4. Discussion

Gait initiation performed in dry-land and water immersion conditions was evaluated using an easy-to-use protocol employing IMUs. Temporal and acceleration-based APA measures were computed in 10 iSCI patients, finding in the aquatic condition an increased first step duration and a decreased RMS acceleration for UT and LT in the ML and AP direction, respectively.

Regarding the temporal-related quantities evaluated with the present protocol, no differences were observed in the duration of the APA between the two conditions (Figure 3). The comparison and the values found here match the ones obtained in previous studies analyzing iSCI patients [19] and healthy populations [27], although evaluated with force platforms. The first step duration was found to be higher in water (see Figure 3) than on dry land, with a median value of 1.44 s (inter-quartile range: 0.42 s) vs. 0.70 s (inter-quartile range: 0.23 s), respectively. Previous studies using center of pressure measurements with a force platform found a similar increase in this phase of gait initiation [19,27]. Specifically, using the center of pressure pattern, Marinho-Buzelli et al. [19,27] were able to distinguish between weight transfer to stance limb (in ML direction) and stepping forward (in AP direction) execution phases: the increased duration of the first step execution was mainly attributed to the latter phase in healthy and iSCI patients [19,27]. In the present analysis, it was not possible to identify the two sub-phases of the first step execution phase due to the technology exploited and the limited number of sensors. A similar analysis would require additional IMUs on the feet or an appropriate validation analysis comparing force platform data of specific pathological populations to potentially include the heel-off event [9]. Even if not considering this distinction, the overall increase in the duration of the execution phase of gait initiation is consistent with the slower speed, the longer stride duration, and the shorter stride length found during walking in water in healthy young adults and the elderly [30,31,32].

Considering the acceleration-related quantities, a significant reduction in the RMS during the first step for the trunk segments was found in both directions across the different trunk levels, i.e., for UT in the ML direction and for LT in the AP direction. Furthermore, a reduction (although not significant) in the RMS metric was also found for LT in the ML direction. Overall, these trends are consistent with previous results in a healthy population [27] and in five cases of iSCI patients [19], where ${\mathrm{RMS}}_{ML,\;UT}$ (median values) were 0.97 and 0.64, ${\mathrm{RMS}}_{AP,\;UT}$ were 0.56 and 0.52, ${\mathrm{RMS}}_{ML,\;LT}$ were 0.99 and 0.61, and ${\mathrm{RMS}}_{AP,\;LT}$ were 0.91 and 0.44 for dry-land and aquatic conditions, respectively. The lower acceleration in water is reasonably explained by the specific physical properties of water (density, hydrostatic pressure, buoyancy, viscosity), and for this reason, aquatic exercises involve not only reduced weight bearing but also an augmented drag. These effects differently influenced the two analyzed segments of the trunk. This could be due to the water level (120 cm depth), so the sensor placed at UT was above the water level (out of water) while walking in the aquatic conditions. Due to this configuration, typically adopted in aquatic therapy, patients experience a different control strategy during gait initiation with a different ratio between the upper and lower part of the trunk in the AP direction. The fact that this aspect did not influence the initial orientation of the trunk in the static standing initial condition was verified. For this reason, only during movement, the different drag experienced by the UT with respect to the LT does have an effect during the execution of rehabilitation motor tasks. Indeed, similar values of ${\mathrm{RMS}}_{AP,\;UT}$ were obtained between healthy [27] and iSCI patients, i.e., 0.37 ± 0.14 (0.68 ± 0.23 in iSCI) and 1.23 ± 0.73 (1.38 ± 0.56 in iSCI), respectively, for dry-land and aquatic conditions (mean ± standard deviation), suggesting that the main effect is represented by the aquatic condition and not by the pathology. Thus, aquatic therapy allows the experience of different control strategies of the trunk segments during gait. Furthermore, it is performed in a more demanding (with higher drag than dry land) but safe condition, as perceived by the iSCI patients using questionnaires [19]. Finally, altering the velocity of movement execution and/or the level of immersion in water would possibly allow us to explore different training conditions for iSCI patients and individuals with different sensorimotor and balance disfunctions.

The present analysis of the gait initiation in iSCI patients was focused on the aquatic activity-based therapy, but it should be considered together with other interventions/therapies. To compensate for loss of function, conventional rehabilitation programs and ongoing care involve physical interventions that often target specific impairments above the level of SCI (i.e., poor strength, low cardiovascular fitness, skill, and joint mobility) [33]. To minimize compensatory mechanisms of functional recovery and provide activation of the neuromuscular system below the level of injury, activity-based therapy interventions are implemented [33]. Such interventions are mainly focused on the recovery of motor and sensory function in the lower limbs, with the aim of walking. However, a meta-analysis with 19 studies find no improvement in terms of independence and function when applied to the lower limbs [33]. More recently, not only overground gait but also other forms of locomotor training such as body weight-supported and robotic-assisted gait were proposed, although no increment in walking speed was found [34]. Future studies should investigate if different activity-based interventions (i.e., aquatic, robotic, body weight-supported, overground) lead to different or similar results in terms of gait initiation parameters.

The simple setup implemented in the present analysis has some limitations. Using a waterproof force platform for gait initiation analysis can allow a complete evaluation of the motor task, as it can estimate the center of pressure pattern, the ground reaction force excursion, and impulse. On the other hand, the high cost and the more complex setup, particularly for the waterproof condition required during aquatic therapy, make this device less suitable than IMUs for routine use during daily therapeutic sessions. Additionally, to further facilitate its use in practice, the setup could be further simplified by reducing the number of sensors. This was investigated in preliminary analyses by exploiting only a single IMU placed on the trunk and by applying an algorithm similar to the one proposed by Gazhit et al. [21]. However, the patterns of raw accelerometer and gyroscope data in the aquatic condition did not allow distinguishing the different phases of APA, as no peaks or features were clearly observable. Therefore, future developments also include adapting existing algorithms and/or designing new algorithms to provide a clear APA detection underwater with a reduced experimental setup. Finally, the findings of the present study should be considered within the characteristics of the patients analyzed. Specifically, the gender was not balanced, although it considers the biased male-to-female ratio found in the epidemiology of traumatic iSCI patient [35].

As a practical implication for aquatic physical therapists, considering the results found, it is suggested to check the level of immersion during the training session, as it could influence the motor control strategy between the upper and lower parts of the trunk. Furthermore, it is proposed to use buoyant supports for the upper limbs to stabilize the parts of the trunk out of the water that does not experience the same drag increase as the ones immersed, particularly in the direction of movement progression, i.e., the AP direction for gait.

## 5. Conclusions

An easy-to-use protocol using four IMUs was implemented to evaluate gait initiation during aquatic therapy sessions in iSCI patients. The temporal and acceleration parameters estimated routinely can evaluate the execution of the motor task performed in dry-land and water conditions, giving important information regarding different control strategies adopted in a different environment.

## Data Availability

Not applicable.
